# Evaluating viral inactivation in the liquid waste stream from a viral total nucleic acid extraction kit for safe disposal

**DOI:** 10.1016/j.bsheal.2025.09.001

**Published:** 2025-09-01

**Authors:** Charles Gan, Melissa Pitton, Lea Caduff, Timothy R. Julian

**Affiliations:** a Eawag, Swiss Federal Institute of Aquatic Science and Technology, Dübendorf 8600, Switzerland; b Swiss Tropical and Public Health Institute, Allschwil 4123, Switzerland; c University of Basel, Basel 4001, Switzerland

**Keywords:** Disinfection, Bacteriophage MS2, Extraction waste, Biosafety guidelines, Wastewater

## Abstract

Safe laboratory processing requires mitigating risks from the release of pathogens into the environment through generated waste streams. This study evaluated the inactivation kinetics of bacteriophage MS2 as a surrogate for infectious viruses in liquid waste produced from total nucleic acid extractions of wastewater. The goal was to determine a waste handling protocol that ensures sufficient viral infectivity loss (i.e., inactivation) for safe disposal. Liquid waste was generated using a viral total nucleic acid extraction kit (Wizard® Enviro Total Nucleic Acid Kit, Promega, The United States of America) containing guanidinium chloride, isopropanol, ethanol, and other residual reagents. MS2 phage was artificially added into liquid waste, and inactivation was monitored over 24 h using double agar layer plaque assays. A one-phase exponential decay model was applied to estimate the time required for safe disposal, showing MS2 inactivation followed an exponential decay pattern, achieving a predicted 6-log_10_ reduction at an average of 2.41 h (145 min), with a 95 % confidence interval of 1.34 h (80 min) to 4.05 h (243 min). However, only the 24-hour holding time was observed to significantly exceed the 6-log_10_ reduction threshold, supporting its recommendation as a conservative and practical holding time after which the waste can be safely disposed of as chemical solvent waste without additional decontamination measures such as autoclaving, as viral infectivity is reduced by at least 6-log_10_.

## 1 Introduction

The extraction of nucleic acids is a cornerstone of molecular biology, enabling researchers to detect, quantify, and characterize viruses in various matrices. Guanidinium hydrochloride-based kits have become ubiquitous for viral ribonucleic acid (RNA) extraction due to their chaotropic nature and ability to lyse cells, denature proteins, and inactivate nucleases [1,2]. These kits are especially indispensable in high-throughput diagnostic and research settings, such as pandemic surveillance and wastewater monitoring programs, where rapid and reliable detection of viral RNA can guide public health interventions [1,[3], [4], [5]].

Despite their widespread use, nucleic acid extraction kits generate substantial liquid waste that presents a significant biosafety and chemical risk. This waste often contains guanidinium chloride alongside flammable solvents such as ethanol and isopropanol, as well as potentially infectious material present in the initial sample. Autoclave steam sterilization is frequently employed as a universal solution to decontaminate biological waste [6,7]. While this approach is effective at sterilizing microbial pathogens, it does not address the risks associated with the presence of volatile and flammable solvents. Furthermore, autoclaving by steam sterilization is inappropriate for nucleic acid extraction kits, as combustion of these solvents during autoclaving poses a safety hazard and is generally advised against at many institutions [[8], [9], [10]].

Given these limitations, alternative waste management protocols are essential to ensure biosafety while mitigating environmental and safety risks. As extraction kits are specifically designed to lyse intact virus and bacterial cells, an important consideration is that the process itself is likely able to inactivate pathogens sufficiently enough to render the resulting waste microbiologically safe. As such, it is reasonable to assume that liquid waste generated during nucleic acid extractions continues to act as a disinfectant and can therefore sufficiently inactivate pathogens on its own. Such an approach would eliminate the need for additional decontamination steps such as autoclaving.

Supporting the assertion that extraction kit waste can act as a disinfectant is wide evidence that some of its main constituents possess virucidal properties. Guanidinium chloride, a common component in lysis buffers used in nucleic acid extraction kits, has been shown to inactivate viruses, with the extent and timeframe of inactivation defined for a few specific workflows and virus models [11,12]. Studies have also shown the usage of similar guanidinium-based products, such as AVL viral lysis buffer (Qiagen, Germany) for inactivation of Ebola [13], usage of lysis buffers for inactivation of severe acute respiratory syndrome coronavirus 2 (SARS-CoV-2) [14,15], or guanidinium thiocyanate for inactivation of poliovirus [16] in a clinical context. Similarly, alcohols such as ethanol and isopropanol used commonly within extraction kits for purification have varying virucidal efficacy depending on target, concentration, and contact time [17]. Given that two of the main substances produced during nucleic acid extraction have some inherent capability of virus inactivation, investigating the time required for complete inactivation could provide insight into whether autoclaving is needed.

In this study, which was motivated by a request to demonstrate safe disposal practices of generated laboratory waste, we focus on the large volume, pooled liquid waste generated during nucleic acid extraction from wastewater using the Wizard Enviro Total Nucleic Acid Kit (cat. no. A2991, Promega, The United States of America [USA]). The waste contains a mixture of guanidinium chloride, isopropanol, ethanol, stabilizers, and biological residues derived from wastewater.

We evaluated the inactivation efficiency of this waste stream using bacteriophage MS2 as a surrogate for viruses that could be pathogenic [18]. MS2 is a non-enveloped virus, a structural characteristic associated with greater resistance to physical and chemical inactivation compared to enveloped viruses. As such, MS2 is frequently used as a conservative surrogate for disinfectant efficacy studies, especially for the more susceptible enveloped viruses such as coronaviruses and influenza viruses, but also for non-enveloped viruses such as Norovirus [19].

Following guidelines from the Biosafety Section of the Office for Waste, Water, Energy, and Air in Zurich, Switzerland, we aimed to determine the minimum holding time of generated waste to achieve a 6-log_10_ reduction of MS2. A 6-log_10_ reduction was chosen by the Biosafety Section as a conservative, but feasible, indicator that is notably above the 4-log_10_ reduction standard recommended by the USA Environmental Protection Agency (EPA) [20]. By addressing this knowledge gap, our findings provide practical evidence for laboratories seeking safe and efficient protocols for managing liquid waste generated during viral nucleic acid extraction of wastewater using the Wizard Enviro Total Nucleic Acid Kit as a commonly used method for direct capture and purification of total nucleic acids from wastewater.

## 2 Materials and methods

### 2.1 Preparation of MS2 stock solution

The bacteriophage MS2 (Deutsche Sammlung von Mikroorganismen und Zellkulturen [DSMZ] 13,767 strain) was propagated using *Escherichia coli* (*E. coli*) (DSMZ 5,695 strain) as the host organism. *E. coli* was cultured overnight at 37 °C with shaking at 220 revolutions per minute (rpm) in 10 mL of autoclaved American Type Culture Collection (ATCC) medium 271, composed of 10 g/L tryptone, 1 g/L yeast extract, 8 g/L sodium chloride, 1 g/L glucose, and 0.3 g/L calcium chloride.

MS2 was propagated in a 0.5 L Erlenmeyer flask by combining 0.1 L of Tris buffer (containing 2.5 g/L tris(hydroxymethyl)aminomethane, 0.6 g/L magnesium sulfate, and adjusted to pH 7.3) with 0.1 L of ATCC medium 271. Streptomycin was incorporated into the mixture to reach a final concentration of 2 mg/L, and magnesium sulfate was added to achieve a final concentration of 0.6 g/L. Next, 2 mL of the log-phase *E. coli* culture and 0.2 mL of an existing MS2 stock solution were added to the Erlenmeyer flask. The flask was gently mixed and incubated at 37 °C for 24 h without shaking. After incubation, the culture was centrifuged at 4,000 *× g* for 15 min, and the supernatant was filtered using 0.22 µm syringe filters (Corning, USA). The filtered solution was further concentrated with an Amicon Ultra-15 centrifugal filter (Merck Millipore, USA) and washed with virus dilution buffer (Nanopure water with 0.78 g/L monosodium phosphate and 0.58 g/L sodium chloride). Final retentate of 1 mL (in virus dilution buffer) was removed and then quantified by performing a ten-fold serial dilution and then applying a spot titration test detailed in the subsequent section “Viral recovery and enumeration”. Retentate was then diluted to approximately a concentration of 10^10^ plaque-forming units per milliliter (PFU/mL), aliquoted and frozen at −20 °C prior to use as the MS2 stock solution.

### 2.2 Waste generation and composition

Liquid waste was collected from nucleic acid extractions of wastewater using the Wizard Enviro Total Nucleic Acid Kit (Promega, USA), following the manufacturer’s instructions. In brief, the extraction involved adding binding buffers containing guanidinium chloride to wastewater samples, followed by isopropanol and ethanol-based washes. The samples, along with the reagents, were passed through a silica membrane using a QIAvac 24 Plus vacuum manifold (Qiagen, Germany), and the resulting waste was collected within the manifold. Waste from multiple extractions was pooled, and 40 mL aliquots were transferred into sterile 50 mL tubes for subsequent spiking experiments.

Estimated proportions of guanidinium chloride and solvents in the extraction waste were calculated based on reagent volumes used per sample and composition data from publicly available safety data sheets. Detailed reagent specifications and references are provided in Table S1.

### 2.3 Sample types and incubation conditions

Positive and negative controls were included in each experiment to ensure the inactivation of the virus is attributable to the composition of the generated waste. Positive controls consisted of the MS2 stock solution, which was measured to determine the amount spiked into each sample. Negative controls were phosphate buffered saline (PBS), also known as isotonic buffer, without MS2 to confirm the absence of contamination. PBS was prepared by dissolving 8.000 g/L of sodium chloride, 0.201 g/L of potassium chloride, 1.135 g/L of disodium phosphate, and 0.272 g/L of monopotassium phosphate, and then adjusting to a pH of 7.4. Additional controls included waste without the addition of MS2 to verify the removal of inhibitory substances, which could impact MS2 infectivity assays and MS2-spiked isotonic buffer to account for virus losses during processing, which equate to measuring the MS2 loss that is not attributable to disinfection potential of the generated waste.

All samples requiring the addition of MS2 (including positive controls, MS2-added negative controls, and the test condition of MS2 in extraction waste) were spiked with MS2 bacteriophage to a final concentration of approximately 10^9^ PFU/mL by adding 1 mL of MS2 stock solution to 40 mL of each matrix. Samples were inverted multiple times and incubated at room temperature (21–23 °C) for 0.33 h, 3 h, 6 h, and 24 h. All conditions (including controls with and without MS2) were conducted in biological triplicate.

### 2.4 Viral recovery and enumeration

To recover MS2 bacteriophage from waste and control matrices, samples were processed using Centricon Plus-70 centrifugal filters with a 10 kDa molecular weight cutoff (Merck Millipore, USA), following the manufacturer’s instructions. Briefly, centrifugal filters were rinsed with 50 mL of PBS (3,000 *× g* for 15 min), 40 mL of sample added (3,000 *× g* for 30 min or until sample is no longer visible in concentrate cup), concentrate cup was then inverted and sample recovered (1,000 *× g* for 3 min).

Viral infectivity of MS2 in the undiluted recovered sample was quantified using a modified double agar layer assay based on USA EPA Method 1602 [21]. Briefly, 0.1 mL of each sample was mixed with 0.2 mL of log-phase *E. coli* culture and 5 mL of molten soft agar (32 g/L tryptic soy agar, 0.6 g/L magnesium sulfate). The mixture was poured over a solidified base layer of tryptic soy agar (15 mL, 40 g/L tryptic soy agar, 0.6 g/L magnesium sulfate) in round Petri dishes and incubated at 37 °C for 12 h. Plaques were counted to calculate PFU/mL.

Since this study focuses on order-of-magnitude, log_10_ reductions, a high-throughput method with lower precision but requiring less material and time was chosen to assess MS2 infectivity at higher PFU/mL concentrations. This approach, known as the “spot titration test” or the “multiple dilution single plate double agar layer (MD/SP DAL)” method, is widely used in phage research for the rapid evaluation of multiple phage types and phage titer simultaneously [22,23]. To implement this, viral enumeration of the sample was conducted simultaneously using the MD/SP DAL method with square tryptic soy agar plates, as described previously [23]. Samples were serially diluted 10-fold across seven dilutions, and 5 µL of each dilution was spotted in technical duplicate onto a layer of soft agar (12 mL) mixed with 0.6 mL of log-phase *E. coli* culture poured over a solidified base layer of tryptic soy agar (30 mL) in square Petri dishes. After incubation at 37 °C for 12 h, plaques were counted at dilutions where individual plaques were distinguishable and then converted to MS2 phage infectivity in PFU/mL. MS2 phage infectivity was reported as an average of the two technical replicates.

### 2.5 Calculation of MS2 log_10_ reduction values

MS2 log_10_ reduction in infectivity, also referred to as MS2 inactivation, was calculated as the negative log_10_ of the ratio of the infectious virus concentration in the waste sample after a specified exposure time to the infectious virus concentration in the MS2-spiked isotonic buffer (used as a control). The resulting log_10_ reduction values were then expressed as the mean ± standard deviation (SD) from triplicate measurements (Tables S2 and S3). To assess the effect of holding time on MS2 inactivation, a one-way ANOVA was conducted. Assumptions of normality and homoscedasticity were tested and confirmed prior to analysis. Post hoc pairwise comparisons were performed using Holm-Šídák’s multiple comparisons test.

## 3 Results

### 3.1 MS2 inactivation in nucleic acid extraction waste

**Fig. 1:**
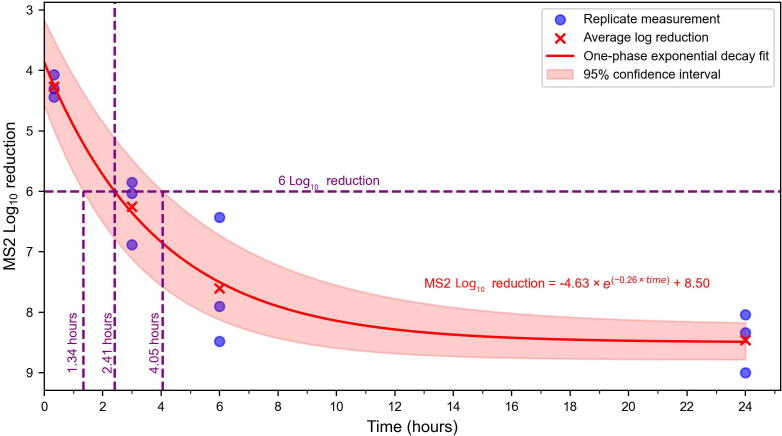
Log_10_ reduction of MS2 infectivity over time in liquid waste. The horizontal axis represents the incubation time (hours), and the vertical axis represents the log_10_ reduction in viral infectivity. Blue circles correspond to individual replicate measurements. Red crosses indicate the empirically-derived average log_10_ reduction at each time point. The red solid curve represents the exponential fit of the data using a one-phase decay model, described by the equation: Log_10_ reduction = −4.63 × *e*^−0.26 ×^*^time^* + 8.50 (*R^2^* > 0.99). The pink shaded area represents the 95 % CI of the fitted curve. The horizontal dashed purple line indicates the 6-log_10_ reduction threshold, while the vertical dashed purple lines mark the estimated mean time point (2.41 h) and the 95 % CI (1.34 to 4.05 h) of when the model predicts the 6-log_10_ reduction is achieved. Abbreviation: CI, confidence interval.

We evaluated MS2 inactivation in liquid waste generated during nucleic acid extractions using the Wizard Enviro Total Nucleic Acid Kit, focusing on achieving a 6-log_10_ reduction in viral infectivity. A total of twelve samples were processed across four time points (0.33, 3, 6, and 24 h) to assess MS2 inactivation in nucleic acid extraction waste. Three biological replicates were performed for each time point. The mean log_10_ reduction in MS2 infectivity increased over time (Fig. 1). At 0.33 h, the mean log_10_ reduction was 4.27 ±0.19, indicating initial inactivation within the first 20 min. By 3 h, the mean log_10_ reduction increased to 6.25 ±0.55, exceeding the 6-log_10_ threshold for effective inactivation defined in this study. At 6 h, the mean log_10_ reduction further increased to 7.60 ±1.06, and at 24 h, the mean log_10_ reduction was 8.46 ±0.49, indicating substantial inactivation.

To characterize the inactivation kinetics and estimate when a 6-log_10_ reduction in MS2 was achieved, the measured data were fit to a one-phase exponential decay curve (Log_10_ reduction = −4.63 × *e*^−0.26 ×^ *^time^* + 8.50; Fig. 1). The model showed excellent fit (R^2^ > 0.99), and replicate measurements closely aligned with the curve. The shaded region surrounding the curve represents the 95 % confidence interval (CI), reflecting the uncertainty in the mean predicted values. Based on the model, the time to reach a mean 6-log_10_ reduction in MS2 was estimated to be 2.41 h (145 min), with a 95 % CI ranging from 1.34 h (80 min) to 4.05 h (243 min).

To further confirm the effect of time on MS2 inactivation, a one-way ANOVA test was performed, and showed a significant effect of time on log_10_ reduction (*P* < 0.001). Holm-Šídák’s multiple comparisons test indicated that later time points (3, 6, 24 h) differed significantly from the first time point of 0.33 h (*P* < 0.05). Additionally, 24 h differed significantly from 3 h (*P* = 0.02). No significant difference was observed between 3 and 6 h (*P* = 0.07) or between 6 and 24 h (*P* = 0.15).

Negative controls (isotonic buffer without MS2) showed no plaque formation, verifying the absence of contamination. Waste samples without MS2 also produced no plaques, demonstrating that residual reagents in the waste did not interfere with infectivity assays or inhibit *E. coli* growth. The MS2-spiked isotonic buffer control showed an expected concentration of 10^9^ PFU/mL, matching the initially spiked amount and confirming that no significant virus loss occurred due to sample processing. These controls validate that observed reductions in MS2 infectivity were attributable to the inactivation potential of the extraction waste.

## 4 Discussion

This study evaluated viral inactivation in liquid waste generated during nucleic acid extractions using the Wizard Enviro Total Nucleic Acid Kit, focusing on achieving a 6-log_10_ reduction in MS2 bacteriophage infectivity. The results demonstrated that a 6-log_10_ reduction in MS2 was estimated to occur at 2.41 h (95 % CI: 1.34–4.05 h) of holding time using a one-phase decay model. However, longer storage should be used to ensure a more robust safety margin. Notably, 24-hour storage is the only measured time point that exceeds the upper bound of the 95 % CI (4.05 h) for achieving a 6-log_10_ reduction and is statistically significantly different from 3 h (which falls below this threshold). This longer time would remain convenient for labs to implement, and the resulting additional holding time could mitigate the risk of incomplete inactivation due to variability in waste composition or processing conditions.

Following sufficient viral inactivation, the waste can be safely classified and disposed of as chemical solvent waste. The key components of the waste, including guanidinium chloride, isopropanol, ethanol, and non-infectious biological residues, no longer pose a substantial biological hazard from viruses after the recommended holding period. This eliminates the need for further disinfection, such as autoclaving, which presents safety risks due to the flammable solvents present in the waste stream. This recommendation aligns with best practices for hazardous waste disposal, emphasizing chemical handling protocols rather than biohazard management once viral infectivity is eliminated.

### 4.1 Implications for laboratory waste management

The findings of this study fill an important gap in laboratory waste management practices. Despite the widespread use of guanidinium chloride-based extraction kits in wastewater monitoring, limited data exist regarding the safety of liquid waste handling and disposal. Autoclaving is often used by default for microbial decontamination but is inappropriate for solvent-rich waste streams. Our results provide an evidence-based framework for managing such waste streams safely, reducing the reliance on autoclaving and its associated hazards while ensuring biosafety.

This study also highlights the role of guanidinium chloride in viral inactivation. Chaotropic agents such as guanidinium chloride are widely recognized for their ability to denature proteins and inactivate nucleic acids. Based on the published safety data sheets for the Wizard Enviro Total Nucleic Acid Kit, we estimated that the liquid waste generated contains 4.7 %–7.1 % guanidinium chloride, which may have contributed to the observed MS2 inactivation. Numerous studies have demonstrated its efficacy for viral inactivation in diagnostic applications [[13], [14], [15], [16],^24^], indicating their potential utility for viral inactivation in other complex waste matrices. However, existing studies on lysis buffers containing chaotropic agents for viral inactivation have also presented variable results, with some reporting effective viral inactivation while others suggesting inconsistent efficacy [25,26].

Alongside guanidinium chloride, alcohols play a significant role in viral inactivation. Based on published safety data sheets for the reagents of the Wizard Enviro Total Nucleic Acid Kit, we estimate that the liquid waste contains approximately 39.5 % isopropanol and 10.0 % ethanol, resulting in a total solvent concentration of 49.5 %, which contributes to its potential for viral inactivation. Previous studies have demonstrated rapid viral inactivation at solvent concentrations between 62.0 %–80.0 %, with complete loss of infectivity occurring within 15 s for some viruses [27]. However, the effectiveness of alcohol-based disinfection varies depending on the solvent type (e.g. ethanol vs. isopropanol), viral species, and initial viral concentration, with optimal inactivation typically occurring at 60.0 %–90.0 % alcohol content [17]. Given that the total solvent concentration in our liquid waste is 49.5 %, it falls below these reported thresholds for rapid viral inactivation of infectivity. This suggests that one potential strategy for reducing the 4-hour inactivation of infectivity time would be to increase the solvent concentration in the waste to 60.0 % or higher, thereby enhancing its disinfectant properties.

By demonstrating that a 6-log_10_ reduction in MS2 infectivity can be achieved within liquid waste containing guanidinium chloride and alcohols within specific percentage ranges, this study provides a foundation and evidence base for the potential of storage of similar waste matrices as sufficient for microbial safety within nucleic acid extraction workflows.

### 4.2 Limitations and future research

While this study offers evidence for safe waste handling, several limitations should be acknowledged. First, the experimental conditions were designed to mimic typical laboratory workflows, but variations in waste composition across different laboratories or extraction protocols may affect viral inactivation kinetics. Use of different kits than the one evaluated here, for example, may lead to differences in the concentration of chaotropic agents or residual biological material that could alter the time required to achieve a 6-log_10_ reduction. Future studies examining the impact of varying guanidine chloride and solvent concentrations in liquid waste could provide valuable insights for optimizing viral inactivation and improving waste management strategies.

We highlight that the conservative suggestion to hold waste for at least 24 h is so effective that it would likely be sufficient for kits with approximately the same composition of liquid waste. Second, MS2 bacteriophage was used as a conservative, non-enveloped surrogate for pathogenic viruses. If there are specific pathogens of concern for a given lab with evidence of decreased susceptibility to inactivation, repeating this experiment to demonstrate sufficient reductions with the target of interest may be important. Finally, this study focused on the inactivation of MS2 infectivity over time at room temperature, and waste treated or stored under different conditions may influence or reduce viral inactivation of infectivity.

### 4.3 Recommendations

Based on these findings, we propose the following recommendations for managing liquid waste from similar nucleic acid extraction workflows with similar composition, large volumes, and waste pooling within manifold (Table S1): First of all, hold liquid waste for a minimum of 4.05 h (upper bound of 95 % CI) after processing to achieve at least 6-log_10_ inactivation of viruses present, providing a safety margin beyond the observed mean 2.41 h timeframe. A conservative 24-hour hold time was found to be the only time point significantly above the 6-log_10_ inactivation threshold. As it is also logistically convenient to store waste for at least 24 h prior to disposal, this duration might be beneficial, as it also provides additional confidence of sufficient inactivation, including potentially less susceptible viruses. Secondly, Classify and dispose of the waste as chemical solvent waste, as it no longer poses a biological hazard but remains a chemical hazard. This avoids the need for autoclaving, which is incompatible with solvent-containing waste streams that present combustion risks. This also avoids the need for the addition of chemical disinfectants, which have associated costs and complicate lab procedures. Thirdly, Incorporate these findings into laboratory biosafety and waste management guidelines to improve consistency and safety in handling liquid waste generated from guanidinium chloride-based extraction protocols.

## 5 Conclusion

This study provides a protocol for evaluating viral inactivation of infectivity in generated laboratory waste and provides evidence supporting the safe handling and disposal of large volume, pooled, liquid waste generated during nucleic acid extractions using a commercially available direct viral total nucleic acid capture and purification kit. By demonstrating the inactivation of MS2 bacteriophage to below the 6-log_10_ threshold, we establish that the waste can be safely managed as chemical solvent waste after sufficient holding time of at least 4.05 h. We note that further inactivation continues to occur after this period, and after 24 h, inactivation is greater than 6-log_10_. These findings support laboratory waste management practices, offering an alternative to adding additional disinfectants or autoclaving waste streams containing guanidinium chloride and solvents for which autoclaving poses a safety hazard due to the presence of flammable and volatile substances.

## Acknowledgements

The authors gratefully acknowledge Katja Zerbe for her insights in the experimental approach and Christoph Ort for his supervision and support throughout this work. This study was supported by the Swiss National Science Foundation (SNSF Sinergia Grant No. CRSII5_205933) and a grant from the Swiss Federal Office of Public Health awarded to Christoph Ort and TRJ.

## Conflcit of interest statement

The authors declare that there are no conflicts of interest.

## Author contributions

**Charles Gan:** Writing – review & editing, Writing – original draft, Visualization, Methodology, Investigation, Formal analysis, Data curation, Conceptualization. **Melissa Pitton:** Writing – review & editing, Methodology, Investigation, Formal analysis, Data curation, Conceptualization. **Lea Caduff:** Writing – review & editing, Methodology, Conceptualization. **Timothy R. Julian:** Writing – review & editing, Supervision, Resources, Methodology, Funding acquisition, Conceptualization.

## Declaration of generative AI and AI-assisted technologies in the writing process

During the preparation of this work the authors used ChatGPT 4.0 in order to check grammar and suggest structure for the abstract, introduction and discussion. After using this tool, the authors reviewed and edited the content as needed and take full responsibility for the content of the published article.
