# Supplementary material for: Evaluating viral inactivation in the liquid waste stream from a viral total nucleic acid extraction kit for safe disposal
Source: Biosaf Health. 2025 Sep 1;7(5):275–80. doi: 10.1016/j.bsheal.2025.09.001 (PMC12624542; doi:10.1016/j.bsheal.2025.09.001)
Supplement: Supplementary Data 1 [file mmc1.docx]

**Supplemental Material**

**Evaluating viral inactivation in the liquid waste stream from a viral total nucleic acid extraction kit for safe disposal**

Charles Gan^a^, Melissa Pitton^a^, Lea Caduff^a^, Timothy R. Julian^a,b,c,*^

^a^ Eawag, Swiss Federal Institute of Aquatic Science and Technology, Dübendorf 8600, Switzerland

^b^ Swiss Tropical and Public Health Institute, Allschwil 4123, Switzerland

^c^ University of Basel, Basel 4001, Switzerland

^*^ **Corresponding author:**

Timothy R. Julian,

*E-mail* address: [tim.julian@eawag.ch](mailto:tim.julian@eawag.ch) (T. R. Julian)

Address: Überlandstrasse 133, Dübendorf 8600, Switzerland

*1 Table*

**Table S1. Estimated chemical composition of pooled extraction waste generated during nucleic acid extraction.** Volumes and chemical percentages were calculated based on the publicly available manufacturer’s protocol and safety data sheets (SDS) for each reagent used in the Promega Wizard Enviro TNA Kit (<https://ch.promega.com/products/nucleic-acid-extraction/viral-rna-extraction-viral-dna-extraction/wastewater-viral-rna-dna-extraction/?tabset0=1&catNum=A2991>, accessed May 23^rd^, 2025). Volume fractions of guanidinium chloride (GuHCl), isopropanol (IPOH), ethanol (ETOH), and other constituents were derived by multiplying each reagent’s composition by the volume added per sample. Ranges are provided where SDS values list a composition range (e.g., 50–75% GuHCl). Final percentages reflect the contribution to the pooled waste of one sample (126.5 mL) used for disinfection testing.

| **Kit Component** | **GuHCl (%)** | **IPOH (%)** | **ETOH (%)** | **Other (%)** | **Amount added per sample (mL)** | **Volume fraction of GuHCl (mL)** | **Volume fraction of IPOH (mL)** | **Volume fraction of ETOH (mL)** | **Volume fraction of other (mL)** |
| --- | --- | --- | --- | --- | --- | --- | --- | --- | --- |
| Protease | 0% | 0% | 0% | 100% | 0.5 | 0 | 0 | 0 | 0.5 |
| Wastewater | 0% | 0% | 0% | 100% | 40 | 0 | 0 | 0 | 40 |
| Binding Buffer D | 50%-75% | 0% | 0% | 25%-50% | 12 | 6-9 | 0 | 0 | 3-6 |
| Binding Buffer E | 0% | 0% | 0% | 100% | 1 | 0 | 0 | 0 | 1 |
| Column Wash 1 | 0% | 40% | 0% | 60% | 5 | 0 | 2 | 0 | 3 |
| Column Wash 2 | 0% | 0% | 63% | 37% | 20 | 0 | 0 | 12.6 | 7.4 |
| Isopropanol | 0% | 100% | 0% | 0% | 48 | 0 | 48 | 0 | 0 |
| Total | 4.7%-7.1% | 39.5% | 10.0% | 43.4%-45.8% | 126.5 | 6-9 | 50 | 12.6 | 54.9-  57.9 |
